# Femoral Revision Total Hip Arthroplasty Performed through the Interval of the Direct Anterior Approach

**DOI:** 10.3390/jcm10020337

**Published:** 2021-01-18

**Authors:** Martin Thaler, Dietmar Dammerer, Michael Ban, Hermann Leitner, Ismail Khosravi, Michael Nogler

**Affiliations:** 1 Department of Orthopaedics and Traumatology, Medical University Innsbruck, Anichstr. 35, 6020 Innsbruck, Austria; dietmar.dammerer@tirol-kliniken.at (D.D.); michael.ban@tirol-kliniken.at (M.B.); ismail.khosravi@gmail.com (I.K.); 2 Institute of Epidemiology, Tirol Kliniken Innsbruck, Anichstraße 35, 6020 Innsbruck, Austria; hermann.leitner@tirol-kliniken.at; 3 Department of Orthopaedics and Traumatology, Experimental Orthopaedics, Medical University Innsbruck, Sonnenburgstr 16, 6020 Innsbruck, Austria; michael.nogler@i-med.ac.at

**Keywords:** direct anterior approach, hip revision, femoral revision, total hip arthroplasty, total hip replacement

## Abstract

Background: we report the clinical outcomes for femoral revision total hip replacement (THR) using the Direct Anterior Approach (DAA) interval. Methods: 149 patients (165 hips) with a mean age of 68.9 years (range, 33.2–91.0 years) and a mean follow-up of 4.2 years (1.1–8.9 years) were included. The indication for revision surgery was aseptic stem loosening in 131 (79.4%) hips, periprosthetic fracture in 29 (17.6%) hips, revision for stem malalignment in one (0.6%) hip, and prosthetic failure in four (2.4%) hips. Results: an endofemoral approach was used for 156 hips, and a Wagner transfemoral osteotomy was used for nine hips. An additional cup revision was done in 52 hips (uncemented cup: *n* = 29; cemented cup: *n* = 21; acetabular cage: *n* = 2). The overall complication rate was 14.5% (24 complications). Ten patients (10 hips) were revised (8 cups, 2 liners, 2 stems) with an average time to revision of 6 months (range, 3–23 months). The median preoperative Western Ontario McMasters Osteoarthritis Score (WOMAC) score was 52.5 (Inter Quartile Range (IQR): 33.3), which improved to 27.2 (IQR: 30) postoperatively (*p* < 0.01). Conclusion: use of the DAA achieved similar results when compared with other surgical approaches in terms of clinical outcomes and complications, including dislocation rate. These results suggest that femoral revision using the DAA interval can be a safe and reliable procedure.

## 1. Introduction

Total hip arthroplasty (THA) is one of the most successful types of surgery performed. It can relieve pain and restore function in patients with diagnoses, such as osteoarthritis of the hip and femoral head necrosis. The number of primary THA procedures is estimated to increase significantly during the coming decades [1,2,3]. Consequently, the number of THA revision procedures will likely increase accordingly. Projection analysis based on registry data indicates that the number of hip revision cases will double by the year 2026 [1].

The Direct Anterior Approach (DAA) in primary THA is a minimally invasive, muscle sparing and internervous approach that may result in better functional recovery and possibly in a higher quality of life [4]. For these reasons, the DAA has steadily become more popular for primary THA. The increasing numbers of THA procedures being conducted using the DAA procedure raises questions regarding the feasibility of performing revision THA through the same interval [5,6]. 

Recent reports on the use of the DAA in cadavers indicated that femoral revision arthroplasty could, in theory, be performed safely through the DAA interval [7]. Small case studies have been published describing the technique of treating periprosthetic femoral fractures by extending the DAA [7]. However, data from larger clinical studies validating the feasibility of using the DAA for femoral revision arthroplasty have not been published. Revision THA is still associated with significantly higher morbidity and complication rates due to increased blood loss, longer hospital stays, longer surgical time, and higher infection, and instability rates [8]. Revision THA can be a very technically demanding procedure, and readmission rates of 10%, reoperation rates of 22%, and postoperative complication rates of 18% have been reported [9,10,11,12]. Due to these and other issues associated with revision surgeries, surgeons may wish to consider reassessing their currently used surgical approaches for femoral revision surgery in order to determine whether or not modifying or changing those surgical approaches may help minimize complications, reduce costs, and improve outcomes. 

The purpose of this study was to evaluate in a large case series the outcomes of femoral stem revision THA conducted through the DAA interval with regard to complication rates, including dislocation rates, and clinical and functional outcomes.

## 2. Materials and Methods

All revision THA procedures in this case series were performed between March 2010 and March 2017 at one institution. All included patients provided signed consent before they participated in the study. The study was conducted in accordance with the Declaration of Helsinki, and the protocol was approved by the Ethics Committee of the Medical University Innsbruck (identification code 1149/2018). Patient demographics were reviewed and included age, gender, body mass index (BMI), date of initial surgery, date of revision surgery, use of proximal or distal extension of the DAA, indication for revision surgery, cut-to-suture time, postoperative follow-up time, and the latest follow-up date. Postoperative complications were examined also, including dislocations, fractures, infections, and nerve lesions. Patients were included only if they underwent femoral revision surgery via the DAA interval. The time to revision and indication for revision were noted for all included patients.

### 2.1. Surgical Technique 

**Figure 1:**
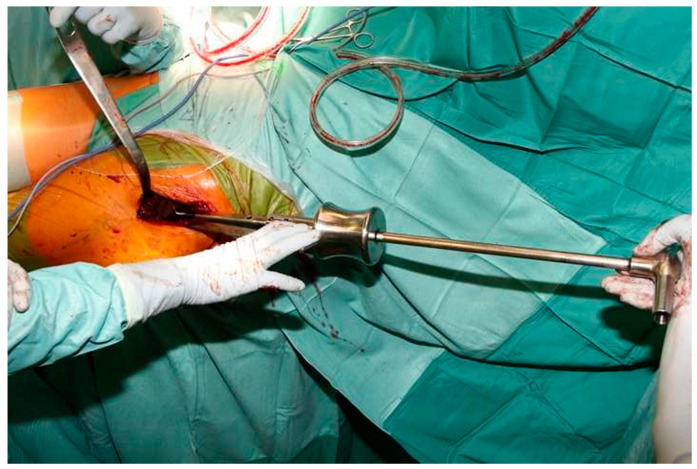
Intraoperative photograph showing straight access to the femur after tensor fascia latae (TFL) release using the Direct Anterior Approach (DAA) interval.

Four surgeons at one hospital performed all procedures using the DAA interval. The technique used for the DAA has been described in detail in a cadaver study [13]. The DAA interval can be extended proximally and distally if needed, which is the case for most revision procedures. Proximally, the approach can be extended to the level of the anterior superior iliac spine (ASIS). The authors prefer not to release the external rotators during stem revision. Instead, they perform a release of the tensor fascia latae (TFL) muscle. The authors recommend incising approximately one-third of the TFL 1–2 cm distally from the ASIS. This release provides straight access to the femur using straight long instruments and implants, and it avoids harming the short external rotators (Figure 1) [7]. 

To access the femoral diaphysis, the skin incision must be extended distally to the appropriate length, which is based on the situation being treated. The authors perform a curved incision starting from the point most distal to the original DAA approach with the cut proceeding laterally and distally in a “lazy S” shape [13]. 

### 2.2. Outcome Assessments 

Radiographs to assess stem alignment were analyzed at the latest radiological follow-uptime point. Heterotopic ossification was classified according to Brooker et al. [14], and radiolucent lines around the femoral component were classified according to Gruen et al. [15]. The Western Ontario McMasters Osteoarthritis Score (WOMAC) [16] was used to determine the function and pain of the patients before surgery and at one-year postoperatively. Failure was defined as femoral revision or removal for any reason, and survivorship was based on the percentage of femoral stems remaining in situ at the latest follow-up time point. Revision was defined as removal of any hip component for any reason. Reoperation was defined as any post-revision intervention involving the operated hip, but without removal of any components. All complications were recorded. 

### 2.3. Statistical Analysis 

Data were analyzed statistically using SPSS (SPSS version 20; IBM Corporation, Armonk, NY, USA). Results for WOMAC scores were analyzed using the Mann–Whitney test. Statistical significance was defined as *p* < 0.05.

## 3. Results

A total of 149 patients (165 hips: 88 males, 77 females) underwent hip revision surgery through the DAA interval. There were 91 right hips and 74 left hips. Sixteen patients underwent bilateral revision THA. Mean patient age at time of revision was 68.9 years (range, 33.2–91.0 years), and the mean BMI was 28.6 (range, 16.9–47.8). Mean patient age at the time of the primary surgery was 63.4 years (range, 30.3–88.7 years). Mean follow-up time after revision surgery was 4.2 years (range, 1.1–8.9 years). During the post-revision follow-up period, six patients (six hips) died for reasons unrelated to the hip or the revision procedure. The revised hips of all six patients were asymptomatic and performing well at the time of the last follow-up visit. Data for those six hips were included in the survival analysis.

The primary THA surgical approach was a direct lateral approach for 105 hips, a posterior approach for one hip, and a DAA for 59 hips. The indication for revision surgery was aseptic stem loosening in 131 (79.4%) hips, periprosthetic fracture in 29 (17.6%) hips, revision for stem malalignment in one hip (0.6%), and failure of the prosthesis in four (2.4%) hips. 83.6% of all revised stems were uncemented. Details regarding the surgical interval and approach extensions are provided in Table 1. 

An endofemoral approach to the femur was used for 156 hips, and a transfemoral approach was used for nine hips. An additional cup revision was done for 52 hips (uncemented cup: *n* = 29; cemented: *n* = 21; cage: *n* = 2). A modular stem was used for 52 hips, and a standard stem was used for 113 hips: CBC (*n* = 63) (Mathys, Bettlach, Switzerland); ABG II (*n* = 6) (Stryker, Kalamazoo, MI, USA); Accolade (*n* = 3) (Stryker); Exeter (*n* = 4) (Stryker); Corail Revision (*n* = 5) (Johnson & Johnson DePuy Synthes, Warsaw, IN, USA); Modular Prothesis (*n* = 49) (Link, Hamburg, Germany); Lubinos SP II (Link) (*n* = 28); RS-ES (*n* = 3) (Implantcast, Buxtehude, Germany). Femoral allograft was used in 10 hips. Mean operative time was 135 min (range, 41–350 min). 

The complication rate for the study cohort was 14.5% (Table 2). Ten (6.1%) of the revised hips required re-revision. The mean time to re-revision was six months (range, 3–23 months). Reasons for re-revision included dislocation (*n* = 6) and infection (*n* = 4). Six dislocated hips required cup revision; four were converted to dual mobility cups, and two were revised to constrained liners. Four hips (2.4%) developed periprosthetic joint infections after the revision THA. These four hips each underwent a 2-stage revision for infection. The cups and stems were explanted, and antibiotic-impregnated spacers were implanted. Subsequent removal of the spacers and implantation of new cups and stems occurred between 3–6 weeks. 

Intraoperative fissures/fractures occurred in four hips (2.4%). Three were lesser trochanter fractures, which were treated with cerclage cable. One patient had a fracture of the greater trochanter and was treated with a claw plate. Six hips that had dislocated underwent closed reduction successfully without recurrent dislocation.

Four (2.4%) patients experienced femoral nerve palsy after the revision THA. In two of the four patients, the femoral lesion had partially resolved by the latest follow-up. Sixteen (9.7%) stems were in a slight varus position, and one (0.6%) stem was in a valgus alignment. At the mean follow-up of 4.2 years, 15 (9.1%) stems had subsided by more than 5 mm. None of those stems were revised, as all patients were pain-free and otherwise asymptomatic. 

**Figure 2:**
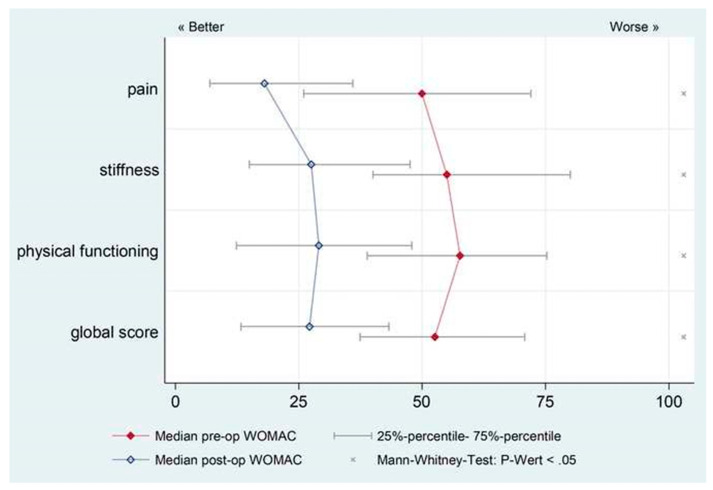
Western Ontario McMasters Osteoarthritis Score (WOMAC) scores showed a significant improvement (*p* < 0.05) postoperatively compared with preoperative scores at a mean follow-up of 4.2 years (range, 1.1–8.9 years). The median preoperative WOMAC score was 52.5 (IQR: 33.3), and the median postoperative WOMAC score was 27.2 (IQR: 30)**.** IQR: Inter Quartile Range.

Radiolucent lines were classified based on Gruen zones [15]. Radiographs of 28 (17.0%) of stems had non-progressive radiolucent lines, mainly at Gruen zones 1, 2, 3, and 7. All of those stems were asymptomatic, and all were well osseointegrated radiographically. In 13 patients, heterotopic ossification was observed at the latest follow-up and was classified according to Brooker et al. [14] as follows: Brooker 1 (*n* = 1); Brooker 2 (*n* = 5); Brooker 3 (*n* = 6); Brooker 4 (*n* = 1). All patients with evidence of heterotopic ossification were asymptomatic. The median preoperative WOMAC score was 52.5 (Inter Quartile Range (IQR): 33.3); it improved significantly (*p* < 0.05) to 27.2 (IQR: 30) postoperatively (Figure 2). No hips were re-revised for mechanical loosening, stem fracture, or subsidence. The percentage of surviving femoral stems at the latest follow-up was 97.3%.

## 4. Discussion

Revision total hip arthroplasty can be a very complex and demanding procedure, particularly on the femoral side. Numerous studies have reported on femoral revision procedures using posterior, lateral, or transtrochanteric approaches [17,18,19]. However, to date, the only studies published about revising a femur through the DAA interval have been cadaver studies. This study reports the clinical results of four surgeons in one orthopedic department who used the DAA interval for femoral revision in 149 patients (165 hips).

The reported incidence of dislocation after primary THA varies from 0.2% to 10%, and from 10% to 28% after revision THA [20,21,22,23]. The dislocation rate of the patients in this case series was 7.3%. In primary THA, the DAA is associated with a lower dislocation rate [24]. It is well known that hip abductor mechanism insufficiency is related strongly to dislocation [25,26,27]. Weakness of the hip abductor muscles may be one of the most important causes of dislocation [28]. In cases of poor exposure, the preferred method of the authors is a release of the tensor muscle in order to protect the external rotators.

The use of larger heads or dual mobility cups in patients requiring revision THA can also decrease the rate of dislocation [29]. Therefore, we cannot conclude from our results that the use of the DAA approach for femoral revisions decreases the risk of dislocation. In general, femoral stem revision is a technically demanding surgical procedure that historically requires an extensive exposure for a controlled removal of previously implanted components, and for management of accompanying bone loss. A repeated insult is often inflicted on the soft tissue envelope, specifically the abductor muscles, resulting in at least a temporary compromise of their function. Therefore, previous hip surgery has been found to be a significant risk factor for hip instability [30]. Four of the 12 patients in this study who dislocated postoperatively were converted to a dual mobility cup, and in another two patients, the liner was changed to a constrained version. Based on prior studies conducted by the authors, the authors now frequently use dual mobility cups in revision cases in order to minimize the risk of dislocation.

In addition to dislocation, infection is one of the leading causes of failure for revision THA [31,32]. The infectiones rate in our study was 2.4%. Rates of infection after revision THA have been reported to range from 0.95% to 22% [9,10,11]. These findings have been attributed to longer surgical time, extensive soft tissue dissection, local tissue scarring, increased dead space, and occult infection [9,10,11]. The infection rate (2.4%), the rate of intraoperative fractures (2.4%), heterotopic ossifications (7.9%), and non-progressive radiolucent lines (17.0%) in this series are well within the range reported in other studies [9]. 

Four (2.4%) patients experienced femoral nerve palsy after the femoral revision surgery. No patients had a peroneal nerve palsy. In addition, no patients complained of meralgic pain postoperatively. However, all patients with a distal extension of the DAA experienced some degree of numbness at the lateral aspect of the operated thigh because branches of the lateral cutaneous femoral nerve must be cut in cases where the approach extension is performed. Therefore, all patients must be told prior to surgery to expect some degree of numbness postoperatively. 

This study has several limitations. One is that it is a case series, and only revisions performed through the DAA interval were evaluated; there was no control group in which another surgical approach was used against to compare outcomes. Another limitation of this study is the minimum follow-up time of one year. The mean follow-up time was 4.2 years (1.1–8.9 years). Data on blood loss, transfusions, and admission to the intensive care unit were not available for this patient cohort. The proportions of revisions presented in the current study varies from that of the published data because only stem revisions were included. All revisions where the femoral stem was left in situ were excluded. In the current study, eight different stem designs were used for revision. This heterogeneity is due to the long inclusion period and the heterogeneity of the patients in the cohort. The heterogeneity of indications (fractures, septic revisions, aseptic loosening) presented in the current study for femoral revisions resulted in a mean stem lifetime of 5.5 years.

**Figure 3:**
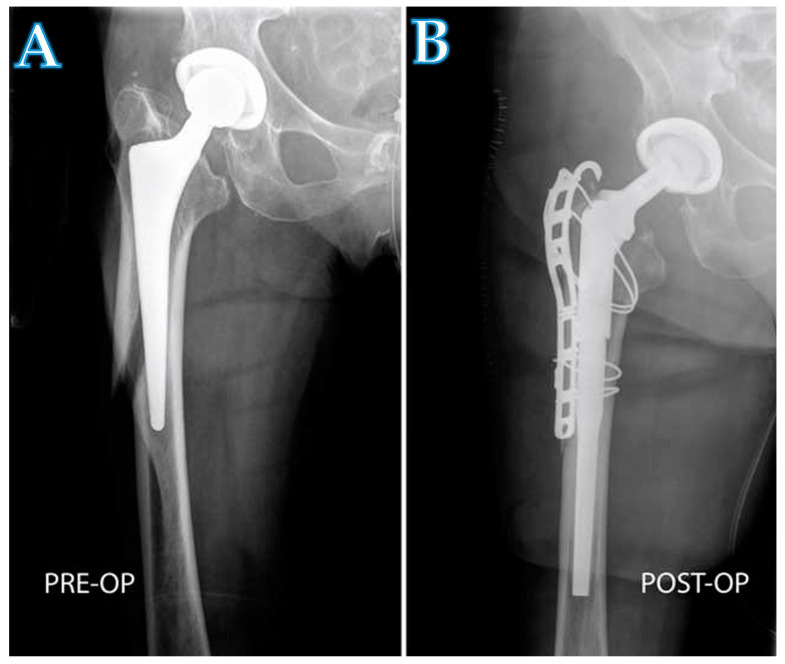
A radiograph showing a patient with a periprosthetic fracture (**A**), and the same patient after being treated with a modular stem (**B**). The implantation of the long modular stem and the reduction of the fracture was performed with a proximal and distal extension of the DAA.

**Figure 4:**
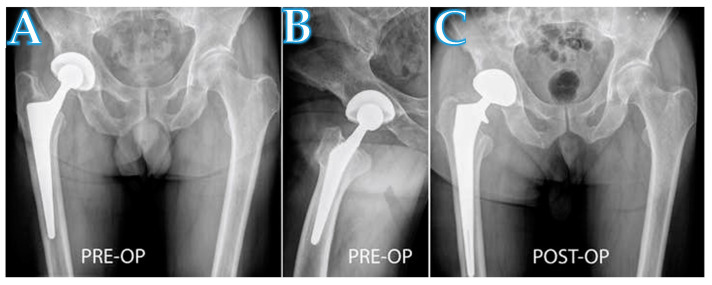
A radiograph showing a patient with aseptic loosening of a femoral stem in the right hip (**A**,**B**). The implantation of the long revision stem was performed with a proximal extension of the DAA (**C**).

The DAA interval for femoral revision arthroplasty is now used in nearly 90% of the revision arthroplasty procedures performed by the practice of authors (Figure 3 and Figure 4). There are three circumstances in which the DAA should not be used as the approach for femoral revision arthroplasties. (1) A draining sinus from another approach. (2) The need to remove a posterior acetabular plate. (3) The need to remove a custom-made implant, which was implanted through another approach. To the knowledge of the authors, this is the first reported series of clinical outcomes and complications regarding patients who have undergone femoral revision THA through the DAA interval. Overall, the complication rate for this cohort (Table 2) and the clinical outcomes (Figure 2) are consistent with rates of other surgical approaches used in a revision arthroplasty setting [33,34]. 

Important considerations for choice of revision approach include the indication for revision, implant type, degree of bone or soft tissue damage, surgeon experience, patient characteristics, such as obesity, type of implant, and fixation [35,36,37]. The authors also believe that both the preferred surgical approach of a surgeon and the level of surgeon training are important contributing factors with regard to clinical outcomes. Over the past several years, many surgeons have been trained in using the DAA. Therefore, this generation of surgeons is accustomed to using the DAA interval. Therefore, performing revisions through that interval will be more or less natural for them. Recent studies have shown the increasing use of the DAA in primary THA in the US and Europe [38,39,40]. Several publications have shown that the DAA provides excellent exposure of the acetabulum. Therefore, revisions of the acetabular component can be achieved easily when using this approach. Although only 20% of surgeons use the DAA for revision cases [38], the results of this study may encourage use of the DAA interval when both components must be addressed surgically. Revision THA through the DAA interval is undoubtedly a technically challenging procedure for untrained surgeons. However, it is anticipated that surgeons who are highly skilled in the use of the DAA for primary procedures can overcome those limitations in order to successfully perform femoral revision arthroplasty procedures.

## 5. Conclusions

New techniques and technologies, such as surgical approaches, minimally invasive instruments, and shorter stems have been introduced for primary THA. Consequently, surgical approaches and traditional techniques should be carefully reconsidered for revision THA as well. The findings of the authors indicate that the complication rate and patient outcomes using the DAA for revision THA correlate with other published data in which other surgical approaches are used. As the number of revision surgeries continues to increase, surgeons should consistently assess and evaluate new ideas and concepts, such as the DAA, which may help them more effectively perform revision THA.

## Figures and Tables

**Table 1:** Interval and approach extensions used for femoral stem revision with the Direct Anterior Approach (DAA).

Approach	N (Hips)	% (Hips)
DAA alone	67	40.7
DAA with tensor release	52	31.5
DAA with distal extension	21	12.7
DAA with distal extension and tensor release	25	15.1
Total	165	100

**Table 2:** Complications.

Complications	N (Hips)	% Hips
Dislocation	12	7.3
Infection	4	2.4
Perioperative fissure/fracture	4	2.4
Nerve palsy	4	2.4
Total	24	14.5

## Data Availability

Data available on request due to restrictions eg privacy or ethical. The data presented in this study are available on request from the corresponding author.
